# A New Geniposidic Acid Derivative Exerts Antiaging Effects through Antioxidative Stress and Autophagy Induction

**DOI:** 10.3390/antiox10060987

**Published:** 2021-06-21

**Authors:** Ying Wang, Yanjun Pan, Yanan Liu, Dejene Disasa, Matsuura Akira, Lan Xiang, Jianhua Qi

**Affiliations:** 1 College of Pharmaceutical Sciences, Zhejiang University, Yu Hang Tang Road 866, Hangzhou 310058, China; 11819026@zju.edu.cn (Y.W.); 21719029@zju.edu.cn (Y.P.); liuyanan1231@zju.edu.cn (Y.L.); 11719053@zju.edu.cn (D.D.); 2 Department of Biology, Graduate School of Science, Chiba University, Chiba 263-8522, Japan; amatsuur@faculty.chiba-u.jp

**Keywords:** geniposidic acid derivatives, iridoid glycoside, antiaging, antioxidative stress, autophagy

## Abstract

Two compounds that can prolong the replicative lifespan of yeast, geniposidic acid (Compound **1**) and geniposide (Compound **2**), were isolated from *Gardenia jasminoides* Ellis. Compared with Compound **1**, Compound **2** was different at C11 and showed better bioactivity. On this basis, seven new geniposidic derivatives (**3**–**9**) were synthesized. Geniposidic 4-isoamyl ester (**8**, GENI), which remarkably prolonged the replicative and chronological lifespans of K6001 yeast at 1 µM, was used as the lead compound. Autophagy and antioxidative stress were examined to clarify the antiaging mechanism of GENI. GENI increased the enzymes activities and gene expression levels of superoxide dismutase (SOD) and reduced the contents of reactive oxygen species (ROS) and malondialdehyde (MDA) to improve the survival rate of yeast under oxidative stress. In addition, GENI did not extend the replicative lifespan of ∆*sod1*, ∆*sod2*, ∆*uth1*, ∆*skn7*, ∆*cat*, and ∆*gpx* mutants with K6001 background. The free green fluorescent protein (GFP) signal from the cleavage of GFP-Atg8 was increased by GENI. The protein level of free GFP showed a considerable increase and was time-dependent. Furthermore, GENI failed to extend the replicative lifespans of ∆*atg32* and ∆*atg2* yeast mutants. These results indicated that antioxidative stress and autophagy induction were involved in the antiaging effect of GENI.

## 1. Introduction

The development of modern medical standards has prolonged the average lifespan of human beings and promoted a substantial increase in the elderly population. According to the United Nations World Population Report in 2019 [1], the world’s elderly population (aged ≥ 65 years) had reached 703 million in 2019 and was expected to increase to 1.5 billion in 2050, accounting for 16% of the world’s total population. The risk of aging-related diseases, such as Alzheimer’s disease, diabetes, and cardiovascular diseases, continues to increase with increased age [2]. Aging has become a risk factor of aging-related diseases. Therefore, the discovery of antiaging molecules and drugs is an important strategy to prevent and treat age-related diseases.

In 1956, Harman proposed the “free radical theory” on aging, which believes that the endogenous oxygen free radicals produced by cells in the body cause damage to cell components and lead to aging and aging-related diseases [3]. ROS in cells is majorly produced in mitochondria during the metabolic process. An excessive level of ROS production causes cellular damage that has an impact on the physiological and pathological processes of the body. ROS is eliminated by the complex antioxidant defense system, including antioxidant and nonantioxidant enzymes. Antioxidant enzymes include SOD, GPx, and CAT. Among them, SOD catalyzes the conversion of superoxide anion into hydrogen peroxide, which is decomposed into water by GPx and CAT to achieve an antioxidant effect [4]. The antioxidant system in the body provides valuable ideas for antiaging research. Under high-concentration oxygen conditions, the overexpression of GPx can improve the antioxidant capacity and prolong the lifespan of fruit flies [5]. Increasing the expression levels of SOD and CAT also improves stress tolerance and prolongs the lifespan of *Drosophila* [6].

Autophagy is an evolutionarily conserved cellular metabolic process. Autophagy can engulf and digest senescent organelles, abnormally folded proteins, or pathogens that invade the body, which are the cells’ defense mechanism [7]. At least 40 autophagy-related genes (*ATG*), which encode proteins involved in autophagy, have been reported [8]. *ATG2* is one of the most important genes involved in autophagy. The protein Atg2 encoded by *ATG2* mediates the direct lipid transfer from the endoplasmic reticulum to the isolation membrane to achieve the expansion of the isolation membrane, thereby forming autophagosomes. In mammals, two Atg2 orthologs, ATG2A and ATG2B, are involved in the process of autophagy [9]. The *ATG32* gene encodes the transmembrane protein Atg32 of the mitochondrial membrane, which is necessary for mitophagy. In yeast, the presence of the Atg32 protein on the outer mitochondrial membrane can identify and target redundant or damaged mitochondria for degradation [10]. Autophagy is a cytoplasmic recovery process that can counteract the accumulation of age-related damaged organelles and proteins, which ultimately improves the metabolic adaptability of cells. Increasing autophagy can remarkably extend the lifespan of mammals [11].

The aging model is an important tool for studying aging, and some of the types of aging models include yeast, nematodes, fruit flies, mice, and zebrafish. Selecting the appropriate model in accordance with its characteristics is the key to conducting research. W303-K6001yeast is a single-celled eukaryote with short growth cycle, easy operation, low cost, and large-scale sample screening [12]. Therefore, the replicative lifespan assay of K6001 is an ideal bioassay system to screen antiaging molecules from natural products. The antiaging effect of resveratrol, which has been the ideal candidate for antiaging drugs, was discovered through the yeast model [13], and its antiaging effect in other biological models has been confirmed [14]. Therefore, using a single-celled yeast as a biological model of antiaging can provide strong support for elucidating the mechanism of aging in higher eukaryotes.

*Gardenia jasminoides* Ellis. (*G. jasminoides*), a traditional Chinese medicine, was one of the first batches of medicine and food resources issued by the Chinese Ministry of Health. *G. jasminoides* has antioxidative, anti-inflammatory, neuroprotective, and antiangiogenesis activities [15]. Furthermore, *G. jasminoides* is rich in chemical components, including iridoids, triterpene saponins, flavonoids, organic acid esters, sterols, and pigment compounds. Iridoids are the main components of *G. jasminoides*. Geniposide, a typical iridoid compound, has a high content of *G. jasminoides* and is one of the effective ingredients. Geniposide can exert many pharmacological effects, such as antidiabetic, antioxidant, neuroprotection, and liver protection properties [16]. In the primary hippocampal neurons of middle-aged Alzheimer’s disease mouse models, geniposide can inhibit the toxic effects of cholinergic defects by increasing choline acetyltransferase activity and reducing acetylcholinesterase activity to play a neuroprotective effect [17].

Two compounds that can prolong the replicative lifespan of K6001 yeast, geniposidic acid (**1**) and geniposide (**2**), were isolated from *G. jasminoides* Ellis. Compound **2** is methylated at the position 11 of Compound **1**, and the antiaging activity was better than **1**, indicating that the esterification at position 11 of geniposidic acid may affect bioactivity. Based on this, we inferred that the structural modification of geniposidic acid may affect its antiaging activity. We designed and synthesized seven antiaging derivatives with new chemical structures (**3**–**9**). Among them, geniposidic 4-isoamyl ester (**8**, GENI) showed the most remarkable effect on prolonging the replicative lifespan of yeast. Thus, GENI was used as the lead compound in the antiaging mechanism study, which involved antioxidative stress and autophagy.

## 2. Materials and Methods

### 2.1. General

*G. jasminoides* was collected from the Taihu Lake in Suzhou, Jiangsu Province, China. The identity of this plant was confirmed by Associate Professor Liurong Chen (College of Pharmaceutical Sciences, Zhejiang University), and a voucher specimen (no. 20190426) was preserved in Zhejiang University, Institute of Materia Medica. Resveratrol (RES) (J&K Scientific Ltd., Beijing, China) was used as a positive control. Silica gel (200–300 mesh, Yantai Research Institute of Chemical Industry, Yantai, China) and reversed-phase C18 (octadecylsilyl, ODS) silica gel (Cosmosil 75 C18-OPN, Nacalai Tesque, Japan) were used for column chromatography. Precoated silica gel (0.25 mm and RP-18 plate (0.25 mm; Merck KGaA, Darmstadt, Germany) were used for TLC. The preparative HPLC was performed on an HPLC system equipped with ELITE P-230 pumps (Dalian Elite Inc., China). NMR spectra were recorded on a Bruker AV III-500 spectrometer (Bruker, Karlsruhe, Germany). High-resolution (HR) ESI-TOF-MS were recorded on an Agilent 6224A LC/MS (Agilent Technologies Inc., Beijing, China).

### 2.2. Isolation of Geniposidic Acid (**1**) and Geniposide (**2**)

The *G. jasminoides* plant material (1.5 kg dry weight) was smashed and extracted using methanol for 24 h under shaking at room temperature. After filtration, the filtrate was subjected to vacuum concentration to obtain 203.2 g extract. The extract was dissolved with water, and *n*-hexane, dichloromethane, ethyl acetate, and *n*-butanol were used to partition the water-soluble substance in sequence. The active *n*-butanol layer (50.0 g) was separated through the ODS open-column chromatography with methanol/water solvent system (20/80, 40/60, 60/40, 80/20, 100/0). Fractions were combined into 10 samples through TLC, and the active Sample 3 (24.1 g) obtained from methanol/water (40/60) elution system was separated again by ODS open column with methanol/water solvent system (30/70, 35/65, 40/60, 45/55, 50/50, 70/30, 100/0) to obtain six samples. About 2 g of Sample 2 (total mass = 14.6 g), which was eluted from the methanol/water (30/70) elution system, was separated by silica open column with a dichloromethane/methanol elution system (100/0, 99/1, 95/5, 90/10, 85/15, 80/20, 70/30, 40/60, 0/100), and 13 samples were obtained. Sample 6 (260 mg) from dichloromethane/methanol (80/20) was purified five times through first HPLC (5C18-AR-II Packed Column [*Φ* = 10 × 250 mm, nacalai tesque], methanol: water = 10:90–50:50, 30 min, 3 mL/min, 210 nm) to obtain the sample (120 mg, *t*_R_ = 10.3 min). The sample was purified thrice by second HPLC (5C18-AR-II Packed Column [*Φ* = 10 × 250 mm, nacalai tesque], acetonitrile: water = 12:88, 3 mL/min, 210 nm) to obtain **1** (60 mg, *t*_R_ = 25.6 min). The structure was identified using spectral analysis, and the ^1^H NMR and HR ESI-TOF-MS spectra data were compared with the literature [18].

About 15 mg of Sample 4 (total mass = 48.1 mg) from dichloromethane/methanol (95/5) was subjected to HPLC purification (5C18-AR-II Packed Column [*Φ* = 10 mm × 250 mm, nacalai tesque], methanol: water = 20:80, 3 mL/min, 210 nm) to obtain **2** (11.2 mg, *t*_R_ = 34.2 min). The structure was identified by spectral analysis, and the ^1^H NMR and HR ESI-TOF-MS spectra data were compared with the literature [18].

### 2.3. Synthesis of Geniposidic Acid Derivatives (**3**–**9**)

**Figure 1:**
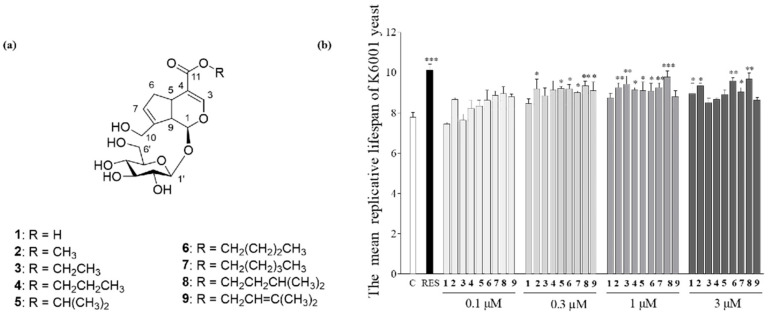
The chemical structure and effect of **1**–**9** on the replicative lifespan of yeasts. (**a**) The structure of **1**–**9**. (**b**) The K6001 yeast replicative lifespan of **1**–**9**. C represents negative control, RES represents positive control (10 µM). Experiments were repeated thrice, and the data are presented as means ± SEM. * *p* < 0.05, ** *p* < 0.01, and *** *p* < 0.001 represent significant difference compared with the control group.

The synthesis of GENI was used as an example. The solution of N,N′-dicyclohexylcarbodiimide (54 mg, 0.26 mmol) and DMAP (8 mg, 0.065 mmol) in dry isoamyl alcohol (4 mL) was added with geniposidic acid (50 mg, 0.13 mmol) at room temperature. The mixture was stirred overnight at 55 °C, and the mixture was concentrated under high vacuum. The residue was extracted with EtOAc (3 × 5 mL). The organic phase was dried over Na_2_SO_4_, filtered, and concentrated. The crude mixture was purified using a silica open column (EtOAc: MeOH = 95:5) and HPLC (5C18-AR-II Packed Column [*Φ* = 10 × 250 mm, nacalai tesque], MeOH: H_2_O = 30:70–50:50, 45 min, 3 mL/min, 210 nm) to obtain GENI (*t*_R_ = 24.0 min). The structures of **3**–**9** (Figure 1a) were determined using spectral analysis. Details are presented in the Supporting Information (S1.1–S1.3, Figures S1 and S2).

### 2.4. Yeast Strains and Lifespan Assay

The K6001 yeast with background W303; wild-type BY4741yeast strain; ∆*sod1*, ∆*sod2,* ∆*uth1*, ∆*skn7*, ∆*cat*, ∆*gpx*, ∆*atg2*, *and* ∆*atg32* mutants with K6001 background; YOM36 and YOM38 containing pR316-GFP-ATG8 plasmid were used. The K6001 yeast strain was obtained from Professor Michael Breitenbach (University of Salzburg, Austria). The K6001 mutant strains of ∆*sod1*, ∆*sod2*, ∆*uth1*, ∆*skn7*, ∆*cat*, ∆*gpx*, ∆*atg2*, ∆*atg32*, BY4741, YOM36, and YOM38 containing pR316-GFP-ATG8 plasmid were provided by Professor Akira Matsuura (Chiba University, Japan). The genotypes of yeast strains and mutants are described in our previous research [19], please see them in Supplementary Table S1.

Replicative lifespan assays were performed following a previous method [20]. In brief, the K6001 yeast strain was collected from a −30 °C freezer after washing with PBS thrice, inoculated in galactose liquid medium (YPG, 1% yeast extract, 2% hipolypeptone, and 3% galactose), and cultured with shaking at 28 °C and 180 rpm for 24 h. After the yeast cells reached the logarithmic growth phase, 4000 yeast cells were inoculated in glucose solid medium (YPD, 2% glucose, 2% hipolypeptone, 1% yeast extract, and 2% agar), which was added with samples in advance and incubated at 28 °C for 48 h. Forty mother cells were randomly selected under the microscope, and surrounding daughter cells were counted. The method for determining conducting the replicative lifespans of ∆*sod1*, ∆*sod2*, ∆*uth1*, ∆*skn7*, ∆*cat*, ∆*gpx*, ∆*atg2*, and ∆*atg32* mutant strains with the background of K6001 was the same as that for K6001 yeast. 

The chronological lifespan assay was conducted as described in our previous study [20]. In brief, the YOM36 yeast strain was collected from a −30 °C freezer and washed with PBS, and about 200 yeast cells were inoculated in glucose solid medium and cultured at 28 °C for 48 h in the stationary phase. A single colony was picked out to a synthetic defined (SD) medium (0.17% yeast nitrogen base without amino acids and ammonium sulfate, 0.5% ammonium sulfate, and 2% glucose) and cultured with shaking at 28 °C and 180 rpm for 24 h. At day 0, yeast cells were inoculated into a new 100 mL SD medium with 0.01 OD_600_ value; treated with 0, 1, and 3 µM GENI; and cultured with shaking at 28 °C and 180 rpm. At day 3, about 200 yeast cells were spread onto glucose solid medium and cultured at 28 °C for 48 h, and the colony forming unit (CFU) of each plate was counted. The CFU of the third day was denoted as 100% survival. These steps were repeated every two days, and the survival rate of each group (CFU per plate/CFU of the same plate on the third day × 100%) was calculated until the survival rate dropped below 5%. 

### 2.5. Evaluation of Antioxidative Stress

The antioxidative stress assay was performed as described in our previous study [20]. For the antioxidant qualitative test, the BY4741 yeast strain in glucose liquid medium with an initial 0.1 OD_600_ value was treated with GENI (0,1, and 3 µM) or RES (positive control, 10 µM) and cultured with shaking at 28 °C and 180 rpm for 24 h. From each group, 5 µL of yeast medium was inoculated in glucose solid medium with 9.5 mM H_2_O_2_ and cultured at 28 °C for 48 or 72 h. The growth of the yeast was observed and recorded. 

For the antioxidant quantitative test, the BY4741 yeast strain in glucose liquid medium treated with samples was cultured with shaking at 28 °C and 160 rpm for 12 h. A blank control medium (0 mM H_2_O_2_ in glucose solid medium) and hydrogen peroxide group medium (5.5 mM H_2_O_2_ in glucose solid medium) were prepared, painted with 200 yeast cells of the different sample groups, and cultured at 28 °C for 48 h. The number of colonies in each group was counted, and the survival rate of each group (number of colonies in the hydrogen peroxide group/number of colonies in the blank control group × 100%) was calculated, plotted, and analyzed.

### 2.6. Measurement of ROS and MDA Levels in Yeast upon Treatment with GENI

ROS was measured as described in our previous study [20]. In brief, the BY4741 yeast strain in glucose liquid medium treated with 0, 1, and 3 µM GENI or 10 µM RES was cultured with shaking at 28 °C and 160 rpm for 12 h. Each group was washed with PBS, added with 2′,7′-dichloro-dihydrofluorescein diacetate fluorescent probes to a final concentration at 10 µM, incubated for 1 h with shaking in the dark, and washed with PBS. The DCF fluorescence was determined using a microplate reader (BioTek, VT, USA) at 488 nm excitation and 525 nm emission wavelengths. 

The MDA was measured as described in our previous study [20]. In brief, the BY4741 yeast strain in glucose liquid medium treated with 0, 1, and 3 µM GENI or 10 µM RES was cultured with shaking at 28 °C and 160 rpm for 12 h, washed with PBS, sonicated on ice for 5 min for protein extraction, and centrifuged at 4 °C and 12,000 rpm for 10 min to obtain the supernatant as protein samples. The protein concentration was determined using the BCA kit (CoWin Biotech, Beijing, China). According to the MDA kit (Nanjing Jiancheng Bioengineering, Nanjing, China) method, the MDA content of each group was determined. Detailed steps are shown in the Supporting Information (S2.1). 

### 2.7. Measurement of SOD, GPx, and CAT Enzymes Activities upon Treatment of GENI in Yeast

The SOD, GPx, and CAT enzymes activities were measured as described in our previous study [20]. The sample processing of BY4741 yeast and the protein extraction were the same as that of MDA measurement in Section 2.6. After determining the concentration of the protein, the activities of SOD, CAT, and GPx were determined using SOD (Nanjing Jiancheng Bioengineering Institute, Nanjing, China), CAT, and GPx (Beyotime Biotechnology Limited Company, Shanghai, China) assay kits, respectively, in accordance with the manufacturer’s instructions. Detailed steps are shown in the Supporting Information (S2.2).

### 2.8. Real-Time Polymerase Chain Reaction (RT-PCR)

The BY4741 yeast strain was collected from a −30 °C freezer and washed with PBS thrice; cells were then diluted in 1 mL PBS and mixed well to add 300 µL to 5 mL glucose liquid medium, and shaking cultured for 24 h at 28 °C with 180 rpm. OD_600_ value was modulated to 0.1, and then treated with GENI (0, 1, and 3 µM) or RES (positive control, 10 µM) and shaking cultured at 28 °C and 180 rpm for 24 h. After the culture, the yeast cells in each group were washed thrice with PBS, and 1 mL PBS was added to mix the yeast, then transferred to the EP tube, supplemented with TES buffer, and vortexed to mix. The total RNA was extracted using the hot phenol method. The reverse transcription method was utilized to synthesize cDNA by using a HiFi-MMLV cDNA Kit (CoWin Biotech, Beijing, China). Quantitative RT-PCR was performed using a CFX96 Touch (Bio-Rad, Hercules, USA) and SYBR Premix EX Taq (Takara, Otsu, Japan), as described in our previous study [21]. The thermal recycling parameters for genes were as follows: SOD1 and SOD2, 95 °C for 2 min followed by 40 cycles of 94 °C for 15 s, 60 °C for 25 s, and 72 °C for 10 s; GPx and CAT, 40 cycles of 95 °C for 15 s and 60 °C for 35 s. The sequences of the primers for RT-PCR were as follows: SOD1, sense 5′-CAC CAT TTT CGT CCG TCT TT-3′ and antisense 5′-TGG TTG TGT CTC TGC TGG TC-3′; SOD2, sense 5′-CTC CGG TCA AAT CAA CGA AT-3′ and antisense 5′-CCT TGG CCA GAA GAT CTG AG-3′; GPx, sense 5′-CGC TCC GTC AAG TAA ACA TAG G-3′ and antisense 5′-GGC CGC TGT TAT TGT TTT GAA C-3′; CAT, sense 5′-TGA CAA ACT CCA CTG GTA ATC C-3′ and antisense 5′-TCC CTG TTG AAA TGA GCC AA-3′; and TUB1, sense 5′-CCA AGG GCT ATT TAC GTG GA-3′ and antisense 5′-GGT GTAATG GCC TCT TGC AT-3′. All results were normalized to those of TUB1. Relative gene expression data were analyzed using the 2^−ΔΔCt^ method. 

### 2.9. Visualization of Autophagy Induced by GENI in Yeast

The YOM38 yeast cells containing the pR316-GFP-Atg8 plasmid yeast strain in glucose liquid medium with initial OD_600_ value of 0.1 were treated with 0, 0.3, 1, and 3 µM GENI or 30 µM RES for 22 h. Cells were washed thrice with PBS and stained with 4′,6-diamidino-2-phenylindole staining solution (20 µg/µL) in the dark. After 10 min, the yeast was washed thrice with PBS and observed using a vertical two-photon confocal fluorescence microscope (Olympus FV1000BX-51, Tokyo, Japan).

### 2.10. Western Blot Analysis

The YOM38 yeast cell containing the pR316-GFP-Atg8 plasmid yeast strain in glucose liquid medium with initial OD_600_ value of 0.1 was treated with 0, 0.3, 1, and 3 µM GENI or 300 µM RES for 22 h. The yeast cells of different groups were collected and sonicated for 5 min. Cell lysates were centrifuged at 12,000× *g* for 15 min to obtain the supernatants for Western blot. Protein concentrations were measured using a BCA protein assay kit (CoWin Biotech, Beijing, China). Western blot was performed as described in our previous study [20]. Approximately 20 µg protein was separated using sodium dodecyl sulfate polyacrylamide gel electrophoresis and transferred onto poly (vinylidene fluoride) membranes. Membranes were incubated with primary antibodies followed by secondary antibodies. The primary antibodies used were as follows: anti-GFP (Medical & Biological Laboratories, Nagoya, Japan) and anti-*β*-actin (CoWin Biotech, Beijing, China). The secondary antibodies used were horseradish peroxidase-linked antirabbit IgGs (CoWin Biotech, Beijing, China). Antigens were visualized using an eECL Western Blot Kit (CoWin Biotech, Beijing, China) and digitized using the ImageJ software (National Institute of Health, Rockville, MD, USA).

### 2.11. Statistical Analysis

Data were evaluated using one-way ANOVA and Tukey’s post hoc test through the GraphPad Prism software (GraphPad Software, CA, USA). The log-rank (Mantel–Cox) test was used for chorological lifespan assay; *p* < 0.05 was considered significant. Each experiment was repeated thrice, and data were expressed as mean ± SEM.

## 3. Results

### 3.1. Structure Identification of Isolated **1** and **2**

The chemical structure of **1** was identified by analyzing HR ESI-MS and ^1^H NMR spectra and comparing with the literature [18]: colorless powder, [α${]}_{D}^{16}$ +15.2 (c 0.6, CH_3_OH), HR ESI-TOF-MS *m/z* 397.1095, calcd. for C_16_H_22_NaO_10_ (M+Na) ^+^ 397.1105. ^1^H NMR (500 MHz, methanol-d_4_): *δ* = 7.51 (1H, s), 5.80 (1H, brs), 5.16 (1H, d, *J* = 7.8 Hz), 4.72 (1H, d, *J* = 7.9 Hz), 4.32 (1H, d, *J* = 14.4 Hz), 4.19 (1H, d, *J* = 14.4 Hz), 3.84 (1H, brd, *J* = 11.8 Hz), 3.64 (1H, m), 3.38 (1H, m), 3.17–3.29 (4H, m), 2.84 (1H, dd, *J* = 8.2, 16.3 Hz), 2.72 (1H, t, *J* = 7.8 Hz), 2.08 (1H, dd, *J* = 8.2, 16.3 Hz).

The chemical structure of **2** was identified by analyzing HR ESI-MS and ^1^H NMR spectra and comparing with the literature [18]: colorless powder, [α${]}_{D}^{16}$ +10.1 (c 2.54, CH_3_OH), HR ESI-TOF-MS *m/z* 411.1269, calcd. for C_17_H_24_NaO_10_ (M+Na) ^+^ 411.1262. ^1^H NMR (500 MHz, methanol-d_4_): *δ* = 7.51 (1H, s), 5.80 (1H, brs), 5.17 (1H, d, *J* = 7.7 Hz), 4.71 (1H, d, *J* = 7.9 Hz), 4.31 (1H, d, *J* = 14.4 Hz), 4.18 (1H, d, *J* = 14.4 Hz), 3.86 (1H, brd, *J* = 12.7 Hz), 3.71 (3H, s), 3.63 (1H, dd, *J* = 5.0, 12.7 Hz), 3.22**–**3.40 (4H, m), 3.18 (1H, m), 2.82 (1H, dd, *J* = 8.2, 16.4 Hz), 2.72 (1H, t, *J* = 7.7 Hz), 2.10 (1H, dd, *J* = 8.2, 16.4 Hz).

### 3.2. Structure Elucidation of Synthesized GENI

The chemical structure of the GENI, which was revealed by analyzing HR ESI-MS, ^1^H NMR, and ^13^C NMR data, was identified to be geniposidic 4-isoamyl ester: colorless powder, [α${]}_{D}^{16}$ +11.1 (c 0.5, CH_3_OH), HR ESI-TOF-MS *m/z* 467.1875, calculated for C_21_H_32_NaO_10_ (M+Na) ^+^ 467.1887. ^1^H NMR (500 MHz, methanol-d_4_): *δ* = 7.50 (1H, s), 5.80 (1H, brs), 5.17 (1H, d, *J* = 7.8 Hz), 4.71 (1H, d, *J* = 7.9 Hz), 4.32 (1H, m), 4.19 (1H, m), 4.16 (2H, m), 3.86 (1H, m), 3.64 (1H, m), 3.38 (1H, m), 3.28 (1H, m), 3.27 (1H, m), 3.23 (1H, m), 3.18 (1H, m), 2.82 (1H, dd, *J* = 8.3, 16.4 Hz), 2.72 (1H, t, *J* = 7.8 Hz), 2.10 (1H, dd, *J* = 8.3, 16.4 Hz), 1.72 (1H, m), 1.57 (2H, m), 0.95 (6H, t, *J* = 6.6 Hz). ^13^C NMR (125 MHz, methanol-d_4_): *δ* = 22.8, 22.8, 26.4, 36.7, 38.6, 39.8, 47.0, 61.4, 62.7, 63.7, 71.5, 74.9, 77.9, 78.4, 98.29, 100.3, 112.8, 128.3, 144.9, 153.2, and 169.2. The ^1^H NMR, HR ESI-MS, and optical rotation data of **3**–**7** and **9** are shown in the Supporting Information (S1.2.1–S1.2.6). The structures of **1**–**9** are shown in Figure 1a.

### 3.3. Structure–Activity Relationship of **1**–**9**

The effects of **1**–**9** on the yeast replicative lifespan at concentrations of 0, 0.1, 0.3, 1, and 3 µM were determined with a K6001 bioactivity system. **1**–**9** can prolong the replicative lifespan of K6001 yeast (Figure 1b). Among them, GENI could significantly prolong the replicative lifespan of yeast at concentrations of 0.3, 1, and 3 µM (*p* < 0.01, *p* < 0.001, and *p* < 0.01) and had the best antiaging activity, revealing that there was esterification at position 11 and that the length and spatial configuration of the introduced carbon chain affected the antiaging effect of yeast. 

### 3.4. Extension of the Replicative Lifespan and Chronological Lifespan of K6001 Yeast by GENI

**Figure 2:**
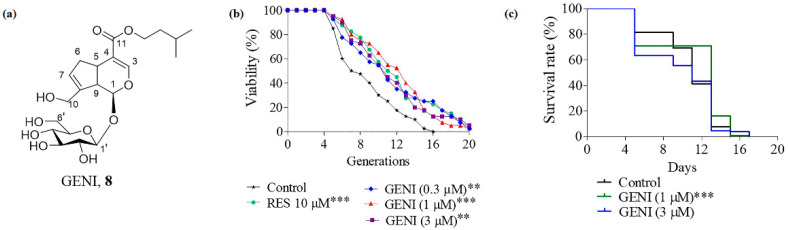
The effect of GENI on the replicative lifespan and chronological lifespan of yeasts. (**a**) The structure of GENI. (**b**) Effect of GENI on the replicative lifespan of K6001 yeast. RES at 10 µM was used as the positive control. (**c**) Effect of GENI on the chronological lifespan of K6001 yeast. Experiments were repeated thrice, and the data are presented as means ± SEM. ** *p* < 0.01 and *** *p* < 0.001 represent significant difference compared with the control group.

As previously mentioned, GENI (Figure 2a) shows a significant increase in the replicative lifespan of K6001 yeast (Figure 2b), and 1 µM is the best concentration (*p* < 0.001). The yeast replicative lifespan of the negative and the positive control groups (10 µM RES) were 7.8 ± 0.54 and 11.05 ± 0.73 (*p* < 0.001), respectively. The average yeast replicative lifespan of 0.3, 1, and 3 µM GENI were 10.40 ± 0.77 (*p* < 0.01), 11.08 ± 0.63 (*p* < 0.001), and 10.50 ± 0.70 (*p* < 0.01). Furthermore, the chronological lifespan assay of YOM36 yeast was performed to evaluate the antiaging activity of GENI; the survival rate of yeast was significantly increased at 1 µM (*p* < 0.001), but there was no change at 3 µM (Figure 2c). These results revealed that GENI exerted antiaging effect on yeast.

### 3.5. Increased Activities of SOD, Reduced Contents of ROS and MDA, and Improved Survival Rate of Yeast under Oxidative Stress by GENI

**Figure 3:**
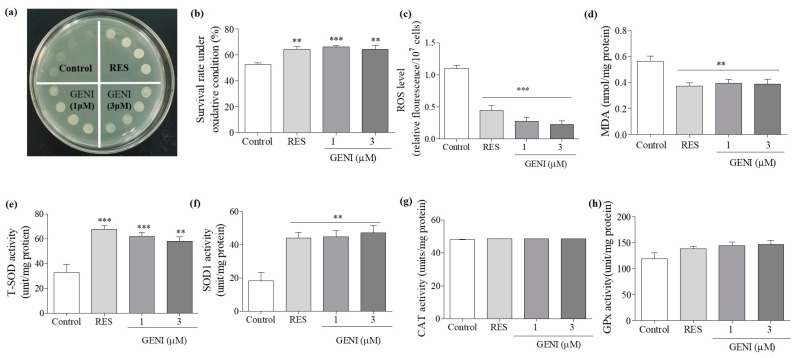
Effect of GENI on the survival rate of yeast under oxidative stress condition and anti-oxidative enzyme activity in yeast. (**a**) Photograph of yeast growth after treatment of GENI under oxidative stress condition induced by H_2_O_2_ at 9.5 m. (**b**) The survival rate changes in yeast under oxidative conditions at 5 mM H_2_O_2_. (**c**,**d**) Effect of GENI on ROS and MDA levels. (**e**–**h**) The changes on T-SOD, SOD1, CAT, and GPx enzyme activities in yeast after treatment of GENI for 24 h. Experiments were repeated thrice, and the data are presented as means ± SEM. ** *p* < 0.01 and *** *p* < 0.001 represent significant difference compared with the control group.

The effects of GENI on the growth of yeast under oxidative stress induced by 9.5 mM H_2_O_2_ are shown in Figure 3a. H_2_O_2_ inhibited the growth of yeast in the negative control group, resulting in a substantial reduction in viable cells. The growth of yeast in 10 µM RES and GENI treatment groups was relatively normal and had evident and dense colonies, indicating that 1 and 3 µM GENI could significantly improve the survival rate of yeast in an environment exposed to oxidative stress (*p* < 0.001 and *p* < 0.01). About 200 yeast cells were spread on a medium containing 0 or 5.5 mM H_2_O_2_ to quantify the viability of yeast, and the survival rate of each group of yeast was calculated by quantifying the number of yeast colonies (Figure 3b). The survival rates of yeast in each group were as follows: negative control: 52.70% ± 1.23%, positive control (10 µM RES): 64.28% ± 2.09% (*p* < 0.001), 1 µM GENI: 66.00% ± 1.09% (*p* < 0.001), 3 µM GENI: 64.18% ± 2.78% (*p* < 0.01). Therefore, GENI showed antiaging activity by inhibiting oxidative stress.

ROS is the main source of free radicals in the body. The effective elimination of ROS is beneficial to protect the cell organelles and molecules from damage, maintain cell homeostasis, and delay aging. ROS can produce the toxic end-product MDA through peroxidation with lipids on the biomembrane, destroy the integrity and normal physiological functions of the biomembrane, further cause protein misfolding and DNA modification, and accelerate aging [4]. Therefore, our research evaluated its antioxidant effect by measuring the effect of GENI on ROS and MDA levels.

The ROS level is shown in Figure 3c. After 24 h of cocultivation with the sample, the average fluorescence values in each group were as follows: negative control: 1.10 ± 0.05, positive control (10 µM RES): 0.44 ± 0.08 (*p* < 0.001), 1 µM GENI: 0.28 ± 0.06 (*p* < 0.001), and 3 µM GENI: 0.23 ± 0.06 (*p* < 0.001). The MDA level is shown in Figure 3d. The average values of MDA for each group were as follows: negative control: 0.56 ± 0.04, positive control (10 µM RES): 0.37 ± 0.03 (*p* < 0.01), 1 µM GENI: 0.39 ± 0.03 (*p* < 0.01), and 3 µM GENI: 0.39 ± 0.04 (*p* < 0.01). GENI could reduce the MDA content in cells at 1 and 3 µM. Based on the above results, GENI exerted antioxidant effects by reducing the levels of ROS and MDA in yeast cells.

The antioxidant enzyme system can effectively eliminate the active oxygen produced by the organism metabolism and reduce the oxidative damage of the organism [4]. Therefore, we evaluated the enzymes activities of T-SOD, SOD1, CAT, and GPx in yeast after 24 h of treatment with GENI at 1 and 3 µM. As shown in Figure 3e–h, the T-SOD andSOD1, enzymes activities were remarkably increased after treatment with 1 and 3 µM GENI. The enzymes activity of GPx also increased to a certain extent, but a significant difference was not observed. However, GENI had no effect on the enzyme activity of CAT. Therefore, GENI exhibited antiaging effects by regulating the T-SOD and SOD1enzymes activities.

### 3.6. Effect of GENI on Gene Expression Levels of SOD1, SOD2, GPx and CAT in Yeast

**Figure 4:**
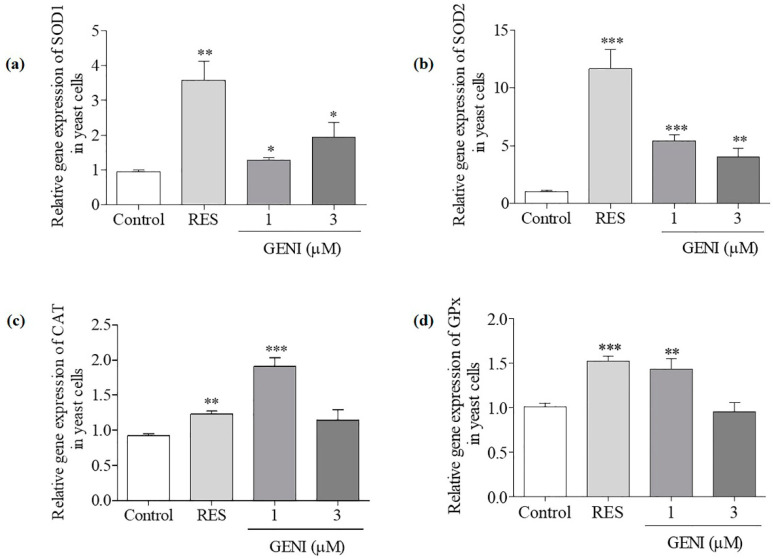
Effect of GENI on *SOD1* (**a**), *SOD2* (**b**), *CAT* (**c**) and *GPx* (**d**) gene expression in BY4741 yeast after treatment of GENI and RES (10 µM). Experiments were repeated thrice, and the data are presented as means ± SEM. * *p* < 0.05, ** *p* < 0.01, and *** *p* < 0.001 represent significant difference compared with the control group.

The changes in *SOD1*, *SOD2*, *GPx*, and *CAT* expression levels of yeast are shown in Figure 4. The gene expression of *SOD1* was significantly increased by 1 and 3 µM GENI (Figure 4a, *p* < 0.05 and *p* < 0.05). The abundance of *SOD2* mRNA was significantly increased after treatment with 1 and 3 µM GENI (Figure 4b, *p* < 0.001 and *p* < 0.01). The significant increase in *CAT* gene expression was only observed in the 1 µM GENI -treated group (Figure 4c, *p* < 0.001). The significant increase in *GPx* expression was only observed in the 1 µM GENI-treated group (Figure 4d, *p* < 0.01). These results indicated that *SOD1*, *SOD2*, *GPx*, and *CAT* genes took important roles in the antiaging effect of GENI.

### 3.7. Involvement of SOD1, SOD2, UTH1, SKN7, CAT, and GPx Genes in the Antiaging Effect of GENI

**Figure 5:**
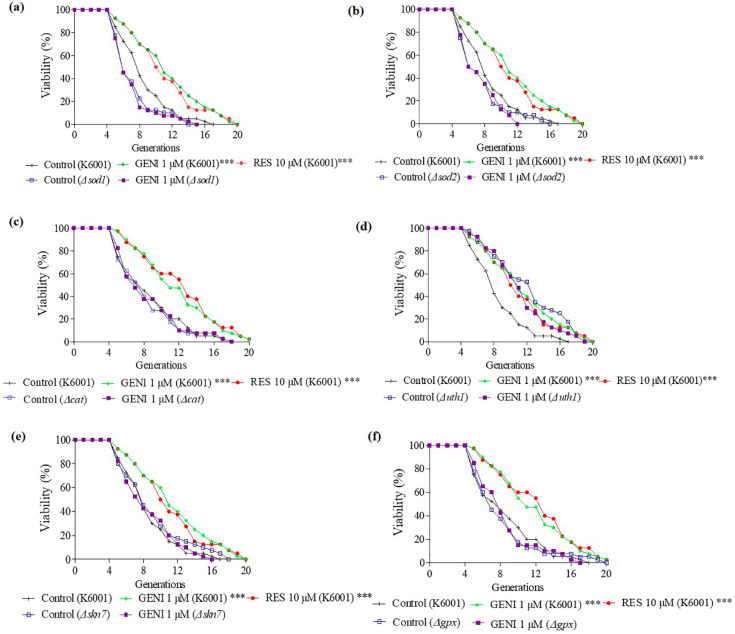
Effect of GENI on the replicative lifespan of K6001 *sod1* (**a**), *sod2* (**b**), cat (**c**), *uth1* (**d**), *skn7* (**e**), and *gpx* (**f**) yeast mutants. Experiments were repeated thrice, and the data are presented as means ± SEM. *** *p* < 0.001 represent significant difference compared with the control group.

*SOD1*, *SOD2*, *UTH1*, *SKN7*, *CAT*, and *GPx* are antioxidative or oxidative-related genes. The replicative lifespan on GENI was analyzed with the *sod1*, *sod2*, *uth1*, *skn7*, cat, and *gpx* mutant strains in the background of K6001, as shown in Figure 5a–f, respectively. The replicative lifespans of K6001 yeast subjected to different treatments were as follows: negative control, 7.58 ± 0.45; positive control (10 µM RES), 10.50 ± 0.60 (*p* < 0.01); and 1 µM GENI, 10.55 ± 0.67 (*p* < 0.001). The replicative lifespans of *sod1* yeast mutant strains subjected to different treatments were as follows: negative control, 6.25 ± 0.38 and 1 µM GENI, 6.13 ± 0.38. The replicative lifespans of *sod2* yeast mutant strains subjected to different treatments were as follows: negative control, 6.68 ± 0.44 and 1 µM GENI, 6.53 ± 0.37. The replicative lifespans of *cat* mutant strains subjected to different treatments were as follows: negative control, 7.18 ± 0.54 and 1 µM GENI, 7.42 ± 0.49. The replicative lifespans of *uth1* mutant strains subjected to different treatments were as follows: negative control, 11.20 ± 0.67 and 1 µM GENI, 10.30 ± 0.57. The replicative lifespans of *skn7* yeast mutant strains subjected to different treatments were as follows: negative control, 8.10 ± 0.60 and 1 µM GENI, 7.63 ± 0.50. The replicative lifespans of *gpx* yeast mutant strains subjected to different treatments were as follows: negative control, 7.18 ± 0.50 and 1 µM GENI: 7.35 ± 0.52. GENI could significantly extend the replicative lifespan of K6001 (*p* < 0.001) but could not prolong the replicative lifespans of *sod1*, *sod2*, *uth1*, *skn7, cat*, and *gpx* mutant strains, indicating that the antiaging effects of GENI were compatible with *SOD1*, *SOD2*, *UTH1*, *SKN7*, *CAT*, and *GPx* genes.

### 3.8. Effects of GENI on Autophagy in Yeast

**Figure 6:**
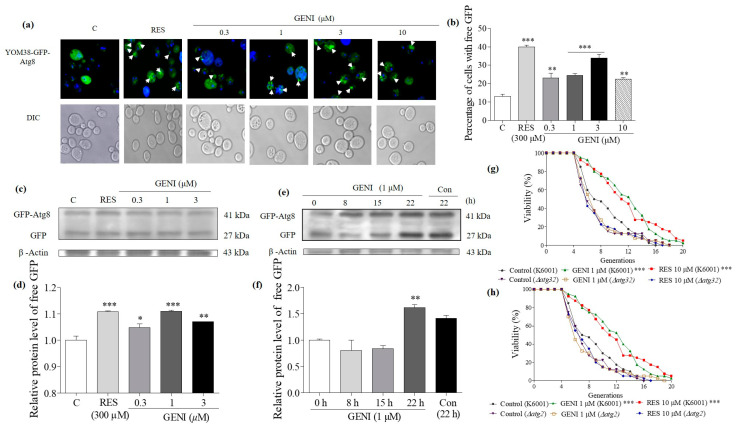
Effect of GENI on autophagy in yeast. (**a**) Fluorescent images of YOM38 yeast containing plasmid pR316-GFP-Atg8 after treatment of RES or GENI observed with a two-photon confocal fluorescent microscope. (**b**) The percentage of YOM38 cells containing free-GFP (green). Seven pictures containing more than 60 cells in each group were used for statistical analysis. (**c**) Western blot analysis of GFP-Atg8 and free GFP in yeast after treatment with RES or GENI for 22 h in SD medium. (**d**) The digital results of (**c**). (**e**) Western blot analysis results for the GFP-Atg8 and free GFP in yeast after treatment with RES or 1 µM GENI in time course. (**f**) The digital results of (**e**). (**g**,**h**) The replicative lifespan of *atg2* and *atg32* of yeast after treatment of RES at 10 µM and GENI at 1 µM. Experiments were repeated thrice, and the data are presented as means ± SEM. * *p* < 0.05, ** *p* < 0.01, and *** *p* < 0.001 represent significant difference compared with the control group.

Autophagy is a degradation process that causes cellular components to cycle into amino acids and other metabolites. Autophagy is closely related to regulating aging [7]. Therefore, the effect of GENI on autophagy was studied. *ATG8*, the LC3 and γ-aminobutyric acid receptor-related protein in mammals, is one of the most important ubiquitin-like systems involved in the formation and maturation of autophagosomes during autophagy [22]. We used the YOM38-GFP-Atg8 yeast strain, which expressed GFP-Atg8 at a physiological level, and detected the free GFP level after GENI treatment by fluorescence microscopy. The fluorescence image is shown in Figure 6a, and the numerical result is shown in Figure 6b. At concentrations of 0, 0.3, 1, 3, and 10 µM, GENI could significantly increase the percentage of cells with free GFP from 13.00% ± 1.29% to 23.00% ± 2.70% (*p* < 0.01), 24.50% ± 0.99% (*p* < 0.001), 34.00% ± 2.03% (*p* < 0.001), and 22.40% ± 0.93% (*p* < 0.01), respectively. The percentage of cells with free GFP treated with 300 µM RES (positive control) increased from 13.00% ± 1.29% to 40.00% ± 1.07% (*p* < 0.001). In addition, Western blot was performed to test the production of free GFP released by autophagy flux into vacuoles. At concentrations of 0, 0.3, 1, and 3 µM, GENI significantly increased the expression level of the free GFP protein (*p* < 0.05, *p* < 0.001, and *p* < 0.01). GENI showed the best effect at 1 µM, which was used to examine the time course of GFP after treatment with GENI (Figure 6c–d and Supporting Information Figure S3a). The released GFP was dependent on time (Figure 6e–f and Supporting Information Figure S3b). *ATG2* and *ATG32* are two important genes involved in autophagy and mitophagy [9,10]. To indicate whether mitophagy is also involved in the anti-aging effect of GENI, we constructed the *atg2* and *atg32* of yeast mutants with K6001 background to measure the changes of replicative lifespan of these mutants. The replicative lifespans of K6001 yeast subjected to different treatments were as follows: negative control, 7.80 ± 0.54; positive control (10 µM RES), 11.05 ± 0.73 (*p* < 0.001); and 1 µM GENI: 11.08 ± 0.64 (*p* < 0.001). The average lifespans of *atg2* in the control, RES, and 10 µM GENI groups were 6.78 ± 0.47, 6.78 ± 0.46, and 6.65 ± 0.53, respectively. The average lifespans of *atg32* in the control, RES, and 10 µM GENI groups were 6.30 ± 0.46, 6.58 ± 0.51, and 6.72 ± 0.50, respectively (Figure 6g–h). These results proved that the autophagy and mitophagy effect of GENI was related to these two genes. GENI showed antiaging effect by inducing autophagy and mitophagy in yeast.

## 4. Discussion

*G. jasminoides* Ellis., a natural product for medicine and food, is widely distributed in China. *G. jasminoides* has the effects of clearing heat, diuresis, and blood cooling and detoxification according to the description of “Shen Nong’s Materia Medica”. However, reports of antiaging molecules in *G. jasminoides* are rare until now. Our research group has long been committed to the chemical and biological research of natural products by conducting structure–activity relationship studies on isolated bioactive molecules to discover leading compounds for Alzheimer’s disease and antiaging drugs. Under the guidance of the K6001 yeast bioassay system, we isolated Compound **1** and **2** from *G. jasminoides*, which can prolong the yeast replicative lifespan. Seven geniposidic derivatives (Figure 1a) were designed and synthesized, and the structure–activity relationship (Figure 1b) was studied. GENI (Figure 2a) was used as a lead compound to study the mechanism of antiaging activity (Figure 2b,c).

Oxidative stress plays a vital role in the aging process, and the antioxidative stress mechanism is a strategy to prevent and treat illnesses related to neurodegenerative diseases [23]. We first studied the effect of GENI on yeast survival rate under oxidative stress conditions, enzymes activity, and related gene expression to understand the mechanism of the anti-aging effect exerted by GENI. The results in Figure 3a–h indicate that GENI exerted antiaging activities by increasing T-SOD and SOD1 enzymes activities and decreasing ROS and MDA levels to improve the survival rate of yeast under oxidative stress. To understand whether these genes and protein involved in antioxidative stress, we investigated these gene expressions and changes of replicative lifespan with PCR and mutants of these genes. The results in Figure 4a–d and Figure 5a–f showed that *SOD1*, *SOD2*, *CAT*, *GPX*, *UTH1*, and *SKN7* were involved in the antiaging effect of GENI. GENI exerted antiaging effects through antioxidative stress.

Autophagy plays an important role in the removal of damaged molecules in the body. Atg8 is a key part of the autophagy mechanism, participates in the entire process of autophagy, and is a biomarker of autophagy in yeast [24]. We first checked whether autophagy was involved in the antiaging effect of GENI. The YOM38-GFP-ATG8 yeast treated with GENI had a remarkable increase in free GFP (Figure 6a–f). Furthermore, no change in the replicative lifespans of ∆*atg2* and ∆*atg32* of yeast (Figure 6g,h) was observed, confirming that autophagy and mitophagy was vital to the antiaging effect of GENI.

For different purposes, we used different concentrations of resveratrol as a positive control in lifespan assay and autophagy detection in the present study. The 10 µM of resveratrol is the best concentration for lifespan assay, and 300 µM of resveratrol is the best concentration for autophagy inducing.

GENI is a new synthetic iridoid derivative. Interestingly, the antiaging active molecules gentiopicroside and amarogentin that we have recently discovered are also iridoid compounds [21,25] that exert considerable antiaging activity through antioxidation or inducing autophagy. Therefore, we speculate that the core structure of iridoids is important for future research on antiaging molecules. In the future, modifications may be made around this structure to improve its bioavailability in animals, which has potential value for the development of innovative drugs.

## 5. Conclusions

The GENI obtained by chemical derivatization from natural products has significant antiaging activity on yeast. It prolongs the replicative and chronological lifespans of yeast by regulating autophagy and antioxidative stress. Geniposidic acid has a high content in plants, a wide range of sources, and low cost. On this basis, many target compounds can be easily synthesized for subsequent research. This research provides an important basis for further research on the mechanism behind the antiaging effects of GENI on different animal models and the development of new drugs for the treatment of aging and neurodegenerative diseases.

## Data Availability

All figures and data used to support this study are included within this article.

## Supplementary Materials

The following are available online at https://www.mdpi.com/article/10.3390/antiox10060987/s1, The experimental details of the synthesized derivatives (**3**–**9**), ^1^H NMR and ^13^C NMR spectrum data, and HR ESI-TOF-MS and optical rotation data are available in the Supporting Information (S1.1–S1.3, Figures S1 and S2). The original data of the western blot of GFP-Atg8 and free GFP induced by GENI are available in the Supporting Information (Figure S3). The genotypes of yeast strains and mutants are described in Supplementary Information, Table S1). The measurement of MDA and the determination of CAT, GPx, and SOD enzyme activities with assay kits are available in the Supplementary Information (S2.1–S2.2).

## Sample Availability

Samples of **1**–**9** are available from the authors.

## Abbreviations

The following abbreviations are used in this manuscript:

GENI	Geniposidic 4-isoamyl ester
CAT	Catalase
SOD	Superoxide dismutase
ROS	Reactive oxygen species
MDA	Malondialdehyde
GPx	Glutathione peroxidase
GFP	Green fluorescent protein
RES	Resveratrol
