# Supplementary material for: A New Geniposidic Acid Derivative Exerts Antiaging Effects through Antioxidative Stress and Autophagy Induction
Source: Antioxidants (Basel). 2021 Jun 21;10(6):987. doi: 10.3390/antiox10060987 (PMC8234659; doi:10.3390/antiox10060987)
Supplement: Supplementary file 1 [file antioxidants-10-00987-s001.zip › antioxidants-1222458-supplementary.pdf]

Supporting Information

Experimental Section

I Chemistry

1.1 General. TLC analyses were done using pre-coated silica gel plates and visualization was done using UV lamp at 254 nm. Preparative HPLC was performed on an HPLC system equipped with ELITE P-230 pumps. NMR spectra were recorded on a Bruker AV III-500 spectrometer. NMR chemical shifts in δ (ppm) were referenced to the solvent peaks of δ_C 77.0 and δ_H 7.26 for $CDCl_3$, δ_C 49.0 and δ_H 3.31 for CD_3OD, and chemical shifts were given in δ (ppm) and signals were described as singlet (s), doublet (d), triplet (t), double doublet (dd), broad (br), and multiplet (m). High-resolution (HR) ESI-TOF-MS was recorded on Agilent 6224A (TOF) LC/MS. All the solvents used were analytical grade.

*1.2 General Procedure for the Synthesis and Structure Elucidation of (**3**) – (**7**), (**9**)*

*1.2.1 Geniposidic 4-ethyl Ester (**3**)*

To a solution of N, N′-dicyclohexylcarbodiimide (DCC) (5.4 mg, 0.026 mmol) and DMAP (0.8 mg, 0.0065 mmol) in dry ethanol (1 mL) was added geniposidic acid (5 mg, 0.013 mmol) at room temperature. The mixture was stirred overnight at room temperature, and then the mixture was concentrated under high vacuum. The residue was extracted with EtOAc (3 × 5 mL). The organic phase was dried over Na_2SO4, filtered, and then concentrated. The crude mixture was purified by column chromatography on silica gel (DCM: MeOH = 95:5), and then purified it by HPLC to obtain a colourless powder (1.0 mg, 19%, t_R=16.3 min,). The HPLC conditions are as follows: Column: 5C18-AR-II Packed Column (Φ 10×250 mm, nacalai tesque); mobile phase system: methanol: water = 30:70–50:50, 40 min; flow rate: 3 mL/min; detection wavelength: 210 nm.

The chemical structure of the **3** was identified to be geniposidic 4-ethyl ester by analyzing HR ESI-MS and ^{1}H NMR data: colorless powder, $[\alpha]_D^{16}$ +9.9 (c 0.5, CH_3OH), HR ESI-TOF-MS *m/z* 425.1415, calcd. for $C_{18}H_{26}NaO_{10}$ $(M+Na)^+$ 425.1418, ^{1}H NMR

(500 MHz, methanol-d_4): δ = 7.51 (1H, s), 5.80 (1H, brs), 5.17 (1H, d, J =7.6 Hz), 4.71 (1H, d, J =7.9 Hz), 4.31 (1H, d, J =14.4 Hz), 4.20 (1H, d, J =14.4 Hz), 4.17 (2H, m), 3.86 (1H, dd, J =1.1, 11.6 Hz), 3.64 (1H, m), 3.38 (1H, m), 3.27 (1H, m), 3.28 (1H, m), 3.23 (1H, m), 3.18 (1H, m), 2.83 (1H, dd, J =8.2, 16.2 Hz), 2.72 (1H, t, J =7.6 Hz), 2.10 (1H, dd, J =8.2, 16.2 Hz), 1.28 (3H, t, J =7.4 Hz).

*1.2.2 Geniposidic 4-propyl Ester (**4**)*

The synthesis method is the same as that of **3**, and the reaction materials are: geniposide acid (10 mg), *n*-propanol (2 mL). The product separation process was the same as that of **3**. The separated target sample was purified by HPLC to obtain a colourless powder (3.6 mg, 32%, t_R=10.7 min). The HPLC conditions are as follows: Column: 5C18-AR-II Packed Column (*Φ* 10×250 mm, nacalai tesque); mobile phase system: methanol: water = 50:50–100:0, 30 min; flow rate: 3 mL/min; Detection wavelength: 210 nm.

The chemical structure of the **4** was identified to be geniposidic 4- propyl ester by analyzing HR ESI-MS and ^{1}H NMR data: colorless powder, $[\alpha]_D^{16}$ +16.7 (c 0.5, CH_3OH), HR ESI-TOF-MS *m/z* 439.1572, calcd. for $C_{19}H_{28}NaO_{10}$ $(M+Na)^+$ 439.1574, ^{1}H NMR (500 MHz, methanol-d_4): δ =7.52 (1H, s), 5.80 (1H, brs), 5.17 (1H, d, J =7.7 Hz), 4.71 (1H, d, J =7.9 Hz), 4.32 (1H, d, J =14.5 Hz), 4.19 (1H, d, J =14.5 Hz), 4.09 (2H, m), 3.86 (1H, dd, J =1.2, 11.9 Hz), 3.64 (1H, m), 3.37(1H, m), 3.28 (1H, m), 3.27 (1H, m), 3.23 (1H, m), 3.19 (1H, m), 2.83 (1H, dd, J =8.2, 16.4 Hz), 2.72 (1H, t, J =7.7 Hz), 2.11 (1H, dd, J =8.2, 16.4 Hz), 1.69 (2H, m), 0.98 (3H, t, J =7.6 Hz).

*1.2.3 Geniposidic 4-isopropyl Ester (**5**)*

The synthesis method is the same as that of **3**, and the reaction materials are: geniposide acid (10 mg), isopropanol (2 mL). The product separation process was the same as that of **3**. The separated target sample was purified by HPLC to obtain a colourless powder (2.3 mg, 21%, t_R=10.7 min). The HPLC conditions are as follows: Column: 5C18-AR-II Packed Column (*Φ* 10×250 mm, nacalai tesque); mobile phase system: methanol: water = 50:50–100:0, 30 min; flow rate: 3 mL/min; detection

wavelength: 210 nm.

The chemical structure of the **5** was identified to be geniposidic 4- isopropyl ester by analyzing HR ESI-MS and ^{1}H NMR data: colorless powder, $[\alpha]_{D}^{16}$ +13.1 (c 0.5, CH_3OH), HR ESI-TOF-MS *m/z* 439.1553, calcd. for $C_{19}H_{28}NaO_{10}$ $(M+Na)^+$ 439.1575, ^{1}H NMR (500 MHz, methanol-d_4): δ = 7.49 (1H, s), 5.80 (1H, brs), 5.15 (1H, d, *J* =7.8 Hz), 5.03 (1H, m), 4.71 (1H, d, *J* =7.9 Hz), 4.32 (1H, d, *J* =14.8 Hz), 4.19 (1H, d, *J* =14.8 Hz), 3.86 (1H, dd, *J* =1.2, 11.9 Hz), 3.64 (1H, m), 3.37 (1H, m), 3.28 (1H, m), 3.27 (1H, m), 3.17 (1H, m), 2.83 (1H, dd, *J* =8.2, 16.3 Hz), 2.71 (1H, t, *J* =7.8 Hz), 2.10 (1H, dd, *J* =8.2, 16.3 Hz), 1.26 (6H, d, *J* =6.2 Hz).

*1.2.4 Geniposidic 4-butyl Ester (**6**)*

The synthesis method is the same as that of **3**, and the reaction materials are: geniposide acid (10 mg), *n*-butanol (2 mL). The product separation process was the same as that of **3**. The separated target sample was purified by HPLC to obtain a colourless powder (5.0 mg, 44%, t_R=12.9 min). The HPLC conditions are as follows: Column: 5C18-AR-II Packed Column (*Φ* 10×250 mm, nacalai tesque); mobile phase system: methanol: water = 50:50–100:0, 30 min; flow rate: 3 mL/min; detection wavelength: 210 nm.

The chemical structure of the **6** was identified to be geniposidic 4- butyl ester by analyzing HR ESI-MS and ^{1}H NMR data: colorless powder, $[\alpha]_{D}^{16}$ +10.4 (c 0.5, CH_3OH), HR ESI-TOF-MS *m/z* 453.1733, calcd. for $C_{29}H_{30}NaO_{10}$ $(M+Na)^+$ 453.1731, ^{1}H NMR (500 MHz, methanol-d_4): δ = 7.51 (1H, s), 5.80 (1H, brs), 5.17 (1H, d, *J* =7.7 Hz), 4.71 (1H, d, *J* =7.9 Hz), 4.32 (1H, d, *J* =14.4 Hz), 4.19 (1H, d, *J* =14.4 Hz), 4.12 (2H, m), 3.86 (1H, d, *J* =11.8 Hz), 3.64 (1H, m), 3.38 (1H, m), 3.28 (1H, m), 3.27 (1H, m), 3.23 (1H, m), 3.18 (1H, m), 2.83 (1H, dd, *J* =8.2, 16.4 Hz), 2.72 (1H, t, *J* =7.7 Hz), 2.10 (1H, dd, *J* =8.2, 16.4 Hz), 1.65 (2H, m), 1.42 (2H, m), 0.96 (3H, t, *J* =7.6 Hz).

1.2.5 Geniposidic 4-amyl Ester (7)

The synthesis method is the same as that of **3**, and the reaction materials are: geniposide acid (10 mg), *n*-pentanol (3 mL). The product separation process was the

same as that of **3**. The separated target sample was purified by HPLC to obtain a colourless powder (3.2 mg, 27%, t_R=23.3 min). The HPLC conditions are as follows: Column: 5C18-AR-II Packed Column (*Φ* 10×250 mm, nacalai tesque); mobile phase system: methanol: water = 50:50–65:35, 40 min; flow rate: 3 mL/min; Detection wavelength: 210 nm.

The chemical structure of the **7** was identified to be geniposidic 4- amyl ester by analyzing HR ESI-MS and ^{1}H NMR data: colorless powder, $[\alpha]_D^{16}$ +14.6 (c 0.5, CH_3OH), HR ESI-TOF-MS *m/z* 467.1877, calcd. for $C_{21}H_{32}NaO_{10}$ $(M+Na)^+$ 467.1888, ^{1}H NMR (500 MHz, methanol-d$_4$): δ = 7.51 (1H, s), 5.80 (1H, brs), 5.17 (1H, d, *J* =7.6 Hz), 4.71 (1H, d, *J* =7.9 Hz), 4.32 (1H, d, *J* =14.4 Hz), 4.19 (1H, d, *J* =14.4 Hz), 4.12 (2H, m), 3.86 (1H, dd, *J* =1.2, 12.0 Hz), 3.64 (1H, m), 3.38 (1H, m), 3.28 (1H, m), 3.27 (1H, m), 3.23 (1H, m), 3.19 (1H, m), 2.83 (1H, dd, *J* =8.2, 16.4 Hz), 2.72 (1H, t, *J* =7.6 Hz), 2.11 (1H, dd, *J* =8.2, 16.4 Hz), 1.67 (2H, m), 1.38 (2H, m), 1.38 (2H, m), 0.93 (3H, t, *J* =7.4 Hz).

*1.2.6 Geniposidic 4-isopentenyl Ester (**9**)*

The synthesis method is the same as that of **3**, and the reaction materials are: geniposide acid (10 mg), prenol (3 mL). The product separation process was the same as that of **3**. The separated target sample was purified by HPLC to obtain a colourless powder (4.2 mg, 36%, t_R=14.1 min). The HPLC conditions are as follows: Column: 5C18-AR-II Packed Column (*Φ* 10×250 mm, nacalai tesque); mobile phase system: methanol: water = 50:50–65:35, 40 min; flow rate: 3 mL/min; Detection wavelength: 210 nm.

The chemical structure of the **9** was identified to be geniposidic 4- isopentenyl ester by analyzing HR ESI-MS and ^{1}H NMR data: colorless powder, $[\alpha]_D^{16}$ +10.9 (c 0.5， CH_3OH), HR ESI-TOF-MS *m/z* 465.1729, calcd. for $C_{21}H_{30}NaO_{10}$ $(M+Na)^+$ 465.1731, ^{1}H NMR (500 MHz, methanol-d$_4$): δ = 7.50 (1H, s), 5.80 (1H, brs), 5.37 (1H, t, *J* =7.2 Hz), 5.17 (1H, d, *J* =7.8 Hz), 4.71 (1H, d, *J* =7.9 Hz), 4.63 (2H, m), 4.31 (1H, d, *J* =14.4 Hz), 4.18 (1H, d, *J* =14.4 Hz), 3.86 (1H, dd, *J* =1.2, 12.0 Hz), 3.64 (1H, m), 3.38 (1H, m), 3.28 (1H, m), 3.27 (1H, m), 3.23 (1H, m), 3.18(1H, m), 2.82 (1H, dd, *J* =8.2, 16.2

Hz), 2.72 (1H, t, *J* =7.8 Hz), 2.10 (1H, dd, *J* =8.2, 16.2 Hz), 1.75 (6H, d, *J* =14.9 Hz).

1.3 The ^{1}H and ^{13}C NMR Spectrum of GENI.

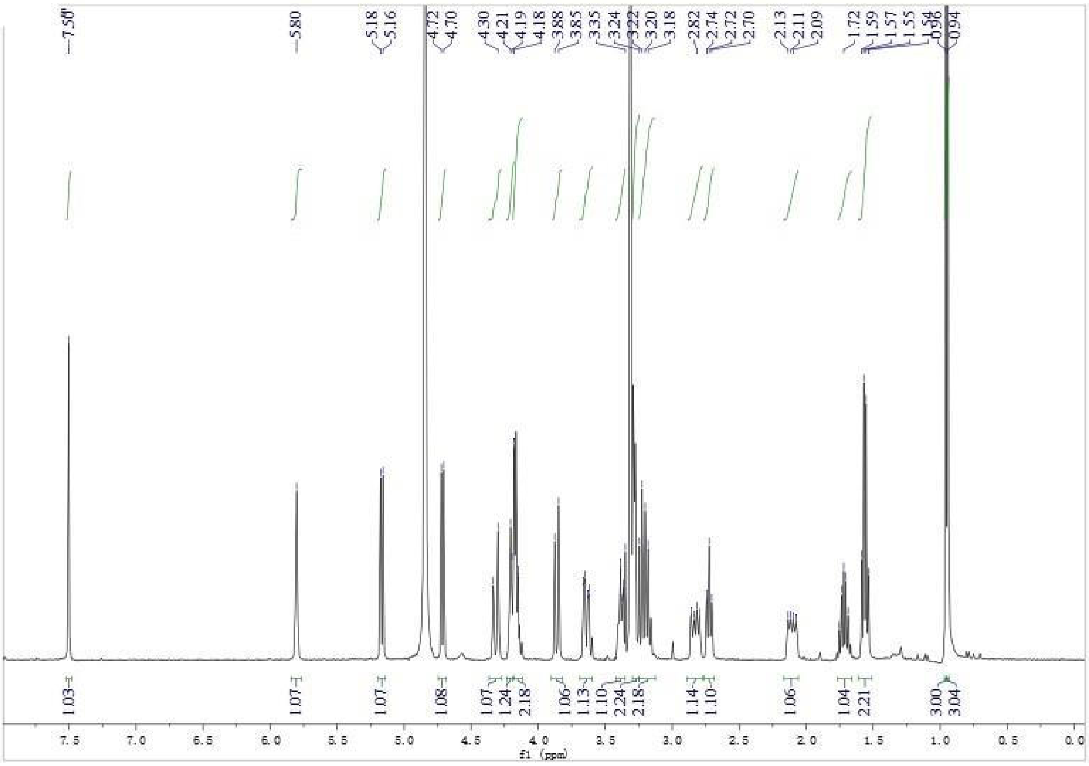

Figure S1. ^{1}H NMR spectrum of GENI (500 MHz, methanol-d_4)

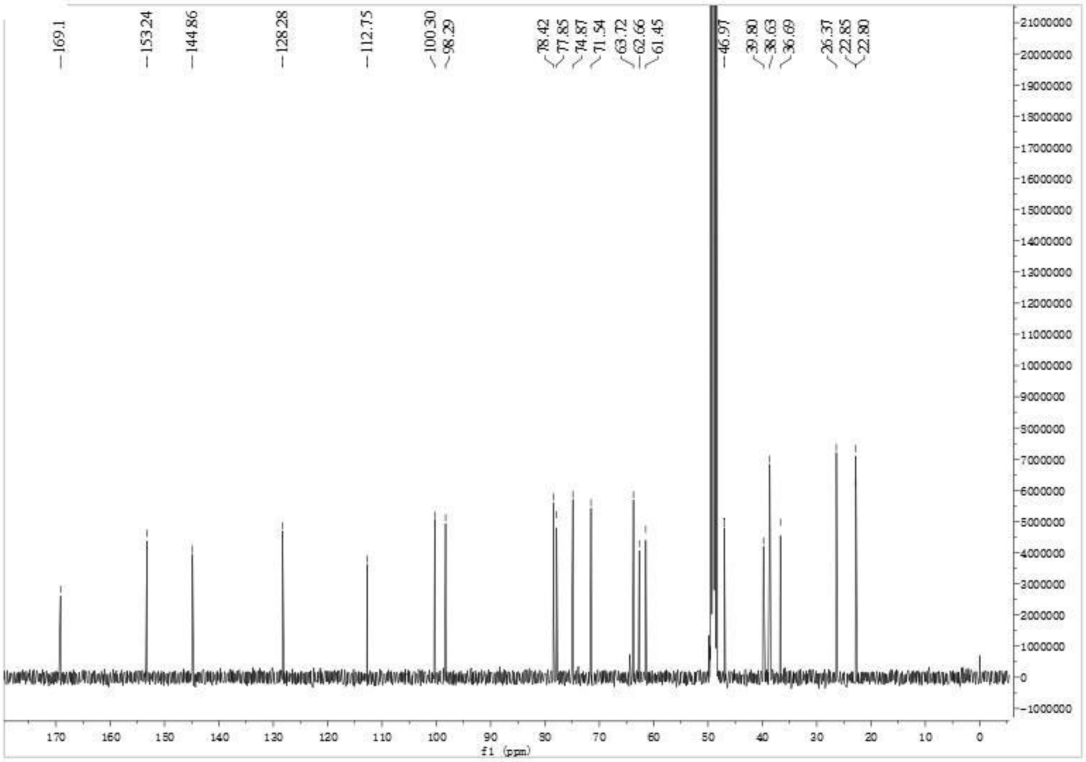

Figure S2. ^{13}C NMR spectrum of GENI (125 MHz, methanol-d_4)

II Biology

2.1 Measurement of MDA with Assay Kits

Set up three sets of blank tube, standard tube, and measuring tube. Prepare reagent I (liquid 20 mL× 1 bottle, stored at room temperature), reagent II (liquid 12 mL× 1 bottle, add 340 mL miliQ water to each bottle and mix well, refrigerate at 4 °C), reagent III (Powder with 60 mL hot miliQ water at 90~100 °C to dissolve it) in advance. Add 100 μL of 10 nmol/ml standard to the standard tube, add 100 μL of absolute ethanol to the blank tube, add 100 μL of sample to the measuring tube. And then add 100 μL of Reagent I to every group and mix well. Then, add 375 μL Reagent II and 125 μL Reagent III (avoid light) and vortex to mix well. The EP tube is tightly sealed with a sealing film, heated in a water bath at 95 °C for 40 minutes, and then cooled with running water. Then centrifuged at 12000 r/min for 10 min and take 200 μL of the

supernatant into a 96-well plate, one tube repeats 2 wells, and the OD value is measured with microplate reader at 532 nm and PBS is used as a blank control.

MDA content = (measuring tube OD value-blank tube OD value) / (standard tube OD value-blank tube OD value) × standard concentration / the protein concentration of samples.

2.2 Measurement of SOD, GPx, and CAT Enzymatic Activities with Assay Kits

For the CAT enzyme activity assay, different concentrations of hydrogen peroxide solution were first taken. Then, a color working solution was added to the CAT enzyme activity assay kit and reacted at 25 °C for 15 min. The absorption value of A520 was measured, and the standard curve of hydrogen peroxide concentration was determined. Afterward, the catalase buffer and 250 mM hydrogen peroxide were added to each sample (5 μg protein). The enzyme reaction termination solution was added after reaction at 25 °C for 1–5 min to terminate the reaction. The color working solution was also added and reacted at 25 °C for 15 min, and the absorption value of A520 was measured.

For the GPx enzyme activity assay, 2 μg of protein of each sample was taken, and the total glutathione enzyme assay kit was used to determine the glutathione enzyme activity. The general protocol is as follows: the GPx detection solution, samples, GPx detection working solution, and peroxide reagent were added in a 96-well plate in proper order. The absorbance value of A340 at every 4 min was measured seven times after mixing.

For the SOD enzyme activity assay, 25 μg of each sample protein was first mixed with reagent VII and reacted for 1 min to remove the Mn-SOD enzyme activity in the samples. The supernatant was then obtained as samples after centrifugation. The reagent I, blank control, samples, and the samples treated by reagent VII were added to the 96-well plate. Afterward, reagents II, III, and IV were added, mixed well, and incubated at 37 °C for 40 min. Finally, the A550 absorbance value of samples was measured.

2.3 The original data of western blot about GFP-Atg8 and free GFP induced by GENI.

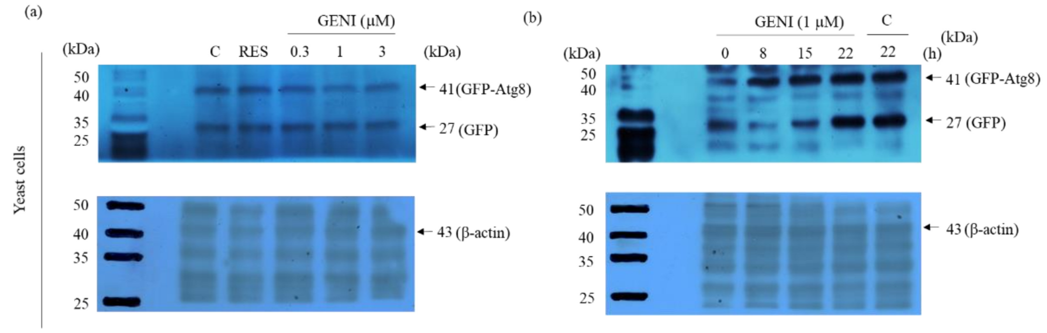

Figure S3. The western blot of GFP-Atg8 and free GFP induced by GENI. (a) Western blot analysis of GFP-Atg8 and free GFP in yeast after treatment with RES or GENI for 22 h in the SD medium. (b) Western blot analysis results for the GFP-Atg8 and free GFP in yeast after treatment with RES or 1 μM GENI in time course.

Supplementary Table1 Yeast Strain used in the present study

**Strains**	**Genotype**	**Source**
K6001	*MATa, ade2-1, trp1-1, can1-100, leu2-3,112, his3-11,15, GAL, psi+, ho::HO::CDC6 (at HO), cdc6::hisG, ura3::URA3 GAL-ubiR-CDC6* (at *URA3)*	Gifted by Professor Michael Breitenbach
Δuth1, Δskn7, Δcat, Δgpx, Δsod1, Δsod2, Δatg2, *Δatg32* of K6001	Replace the *UTH1* gene, *SKN7* gene*, SOD1* gene, *SOD2* gene, *ATG2* gene, and *ATG32* gene in K6001 with kanamycin gene, respectively	Constructed by Professor Akira Matsuura
BY4741	*MATa, his3Δ1, leu2Δ0, met15Δ0, ura3Δ0*	Constructed by Professor Akira Matsuura
YOM36	Prototrophic derivative of BY4742 (*MATα, his3Δ1, leu2Δ0,lys2Δ0, ura3Δ0*)	Gifted by Professor Akira Matsuura
YOM38 containing plasmid pR316-GFP-ATG8	Prototrophic derivative of BY4742 (*MATα, his3Δ1, leu2Δ0, lys2Δ0*) containing plasmid pR316-GFP-ATG8	Constructed by Professor Akira Matsuura
